# Gradient-based autonomous obstacle avoidance trajectory planning for B-spline UAVs

**DOI:** 10.1038/s41598-024-65463-w

**Published:** 2024-06-24

**Authors:** Wei Sun, Pengxiang Sun, Wei Ding, Jingang Zhao, Yadan Li

**Affiliations:** School of Geomatics, Liaoning Technical University, Fuxin, 12300 Liaoning China

**Keywords:** B-spline, Gradient descent, L-BSGF, Trajectory planning, Electrical and electronic engineering, Mechanical engineering

## Abstract

Unmanned aerial vehicles (UAVs) have become the focus of current research because of their practicability in various scenarios. However, current local path planning methods often result in trajectories with numerous sharp or inflection points, which are not ideal for smooth UAV flight. This paper introduces a UAV path planning approach based on distance gradients. The key improvements include generating collision-free paths using collision information from initial trajectories and obstacles. Then, collision-free paths are subsequently optimized using distance gradient information. Additionally, a trajectory time adjustment method is proposed to ensure the feasibility and safety of the trajectory while prioritizing smoothness. The Limited-memory BFGS algorithm is employed to efficiently solve optimal local paths, with the ability to quickly restart the trajectory optimization program. The effectiveness of the proposed method is validated in the Robot Operating System simulation environment, demonstrating its ability to meet trajectory planning requirements for UAVs in complex unknown environments with high dynamics. Moreover, it surpasses traditional UAV trajectory planning methods in terms of solution speed, trajectory length, and data volume.

## Introduction

With the rapid development of artificial intelligence technology and machinery manufacturing, micro UAVs have expanded their capabilities and are now being utilized in various scenarios, not only in the military but also in commercial applications, including environmental detection, fire rescue, road guidance, and short-range transportation^1,2^. However, challenges such as limited load capacity and range continue to hinder the advancement of micro UAVs. Additionally, researchers are faced with the issues of limited perception ability and computational load of micro UAVs towards the external environment^3^.

In response to these problems, experts and scholars have proposed various methods for solving UAV trajectories. One of the traditional path planning algorithms is the artificial potential field method, which has a simple structure and strong real-time performance. However, this method tends to result in local optimal solutions. To address this issue, many scholars have made improvements to the artificial potential field method. Fang et al. introduced a Piecewise-potential-field (PPF) based UAV formation trajectory planning method^4^. This method utilizes a suitable PPF function to avoid local optimal solutions while still satisfying the motion constraints. Li et al. proposed an artificial potential field-based particle swarm algorithm, which generates robot planning paths by adjusting the inertia weight parameter and ranking the position vector of particles^5^.

With the advancement of artificial intelligence, intelligent bionics algorithms have emerged as the principal path planning algorithms^6–8^. The graph search algorithm uses multi-source sensor information to search feasible path options at high frequency and finally calculates the optimal path from the starting point to the destination through an iterative process. Scholars have used intelligent bionic algorithms to enhance traditional graph search algorithms like Dijkstra and A*, significantly boosting the efficiency of local path planning. Zhang et al. proposed the A*-ACO algorithm by fusing the A* algorithm and ant colony optimization (ACO) to improve the blindness of the initial search and the convergence speed of the ant colony algorithm, but the A*-ACO algorithm may not be able to find the optimal path when facing complex situations^9^.

Model predictive control is the mainstream UAV control method. Chai et al. utilized deep neural networks trained with optimal trajectories generated by fuzzy multi-target transcription methods^10^. The trained neural network serves as a UAV command generator to ensure the real-time generation of optimal trajectories. Furthermore, they developed a centralized robust model predictive control algorithm^11^. This centralized control structure simplifies controller development and parameter tuning while guaranteeing a stable and convergent attitude-tracking process through nonlinear feedback rules and stringent constraints. Lindqvist B et al. proposed a nonlinear model predictive control algorithm to predict the future position of obstacles by parameterizing obstacle trajectory so as to conduct autonomous navigation experiments of UAVs^12^. In addition, a new nonlinear model predictive control framework is proposed^13^. Based on this framework, the UAV conforms to the dynamic characteristics of the UAV and effectively solves the problem of UAV estimation drift.

The gradient-based motion planning method, widely used in UAV trajectory optimization, involves transforming the UAV trajectory planning problem into an unconstrained nonlinear optimization problem^14,15^. Ratliffet et al. introduced the Euclidean Signed Distance Field (ESDF) into the field of robot motion planning^16^, using ESDF data to optimally configure the robot's surroundings, which in turn is useful for generating collision-free paths for the robot.

The gradient motion planning method parameterizes trajectory planning^17^. However, due to the need for the UAV to maintain a high-speed motion state, the computational burden imposed by the optimal configuration of the surrounding environment is too heavy for the UAV to handle. As a result, continuous solving of the obstacle avoidance problem becomes challenging, leading to a low success rate^18,19^. To address issues such as excessive data volume in the gradient motion planning method, the B-spline generation method is introduced for trajectory initialization^20,21^. This method helps solve the challenging problem of trajectory solving, focusing on the smoothness and glossiness of the trajectory rather than considering collision problems during the initialization process. The feasibility of the trajectory is ensured by the convex packet property of the B-spline. This property allows for a quick trajectory initialization while avoiding the need for numerous calculations to establish collision-free sections using conventional methods like the potential field method. After initializing, only the gradient data of the ESDF within a narrow range around the initial trajectory are collected^22^. Meanwhile, the initial trajectory is continuously collision-tested and iteratively optimized^23,24^. If a collision is detected, the trajectory undergoes obstacle avoidance modification to generate a collision-free trajectory. This approach significantly reduces the computation of data and enhances the solution speed of trajectory optimization.

The primary objective of the algorithm proposed in this study is to streamline the process of initializing and optimizing trajectories for UAVs. The algorithm aims to enhance the smoothness of trajectories while prioritizing feasibility and safety, making them more suitable for high-speed UAV flight to prevent waste of power and time.

The key contributions of this paper include: (1) introducing a method for UAV trajectory initialization, where B-spline curve control points are chosen through polynomial fitting and connected to ensure the shortest trajectory in obstacle-free scenarios. When obstacles are present, B-spline curves are created based on control points and basis functions, with trajectory obstacle avoidance optimization achieved through the use of the convex hull property. (2) Solving the UAV obstacle avoidance problem using the repulsive force algorithm, proposing a lightweight UAV trajectory evaluation function, and utilizing the L-BSGF algorithm for fast trajectory optimization and quick program restart after planning failure. The proposed algorithm is verified in the Robot Operating System (ROS) environment.

The rest of this paper is organized as follows. Section II focuses on the initialization and obstacle avoidance optimization of the UAV trajectory. Section III simulates and validates the B-spline-based method of UAV trajectory generation in the ROS environment. Section IV draws the conclusions of the paper.

## Trajectory initialization

The UAV trajectory should be collision-free, with the shortest path length, and conform to the smooth curve of UAV dynamics. The B-spline is capable of generating a curve that precisely satisfies these conditions in the given environment. The expression of the B-spline is shown in Eq. (1).

1 $$P(u) = \sum\limits_{i = 0}^{n} {C_{i} B_{i,k} (u)\quad \quad u \in \left[ {u_{k - 1} ,u_{n = 1} } \right]}$$

where $C_{i}$ represents the control point, $B_{i,k}$ represents the B-spline basis function of order $K$, and $u$ represents the node vector. To simplify B-spline generation and improve the efficiency of path generation, the optimal 4th-order basis functions are used to generate B-spline curves. The curve segmentation generates each B-spline trajectory by four control points and four B-spline basis functions calculated by Eq. (2)

2 $$\left\{ \begin{gathered} f_{1} (s) = \frac{1}{6}(1 - s)^{3} \hfill \\ f_{2} (s) = \frac{1}{6}(3s^{3} - 6s^{2} + 4) \hfill \\ f_{3} (s) = \frac{1}{6}( - 3s^{3} + 3s^{2} + 3s + 1) \hfill \\ f_{4} (s) = \frac{1}{6}s^{3} \hfill \\ \end{gathered} \right.$$

where s from 0 to 1 represents the normalized distance, and each segment of the trajectory is generated as shown in Eq. (3).

3 $$P_{i} \left( s \right) = f_{1} \left( s \right)C_{1} + f_{2} \left( s \right)C_{2} + f_{3} \left( s \right)C_{3} + f_{4} \left( s \right)C_{4}$$

where $C_{1}$, $C_{2}$, $C_{3}$, $C_{4}$ is the four control points of the trajectory and $P_{i}$ belongs to $R^{3}$. The complete trajectory of the UAV is stitched by each segment of the trajectory, and the next segment is generated by the control points $C_{2}$, $C_{3}$, $C_{4}$, $C_{5}$. When there are *N* control points, *N*-3 segments of UAV trajectories are generated. The velocity $V\left( s \right)$ and acceleration $A\left( s \right)$ of the position curve can be deduced from 3.

4 $$V(s) = \frac{\partial P(s)}{{\partial s}} = \left[ \begin{gathered} V_{x} (s) \hfill \\ V_{y} (s) \hfill \\ V_{z} (s) \hfill \\ \end{gathered} \right]$$

5 $$A(s) = \frac{{\partial^{2} P(s)}}{{\partial^{2} s}} = \left[ \begin{gathered} a_{x} (s) \hfill \\ a_{y} (s) \hfill \\ a_{z} (s) \hfill \\ \end{gathered} \right]$$

Since the control points of the trajectory are immobile, differentiating the trajectory is differentiating the spline function. Then the velocity spline functions* V*_1_,* V*_2_,* V*_3_,* V*_4_, and the acceleration spline functions* a*_*1*_*, a*_*2*_*, a*_*3*_*, a*_*4*_ can be obtained.

6 $$\left\{ \begin{gathered} v_{1} \left( s \right) = - \left( {1 - s} \right)^{2} /2 \hfill \\ v_{2} \left( s \right) = \left( {3s^{2} /3 - 2s} \right) \hfill \\ v_{3} \left( s \right) = - 3s^{2} /3 + s + 1/2 \hfill \\ v_{4} \left( s \right) = s^{2} /2 \hfill \\ \end{gathered} \right.$$

7 $$\left\{ \begin{gathered} a_{1} \left( s \right) = 1 - s \hfill \\ a_{2} \left( s \right) = 3s - 2 \hfill \\ a_{3} \left( s \right) = 1 - 3s \hfill \\ a_{4} \left( s \right) = s \hfill \\ \end{gathered} \right.$$

Therefore, both the velocity and acceleration of the B-spline trajectory $P_{i}$ are functions of the parameter *S*, as shown in Eqs. (8) and (9).

8 $$V\left( s \right) = v_{1} (s)C_{1} + v_{2} (s)C_{2} + v_{3} (s)C_{3} + v_{4} (s)C_{4}$$

9 $$A\left( s \right) = a_{1} (s)C_{1} + a_{2} (s)C_{2} + a_{3} (s)C_{3} + a_{4} (s)C_{4}$$

This shows that the end point of the former curve and the start point of the latter curve are equal in position, velocity, and acceleration, which ensures that the B-spline trajectory is smooth and continuous. It is also assumed that additional control points are added to verify that the trajectory passes through the start point and end point. We expand the starting point $C_{1}$ as $c_{0}$, $c_{1}$, and $c_{2}$, where $c_{1}$ is the origin $C_{1}$, $c_{0}$ and $c_{2}$ as in Eq. (10).

10 $$\left\{ {\begin{array}{*{20}c} {c_{0} = c_{1} - v_{1} L} \\ {c_{2} = c_{1} - v_{1} L} \\ \end{array} } \right.$$

where $v_{1}$ represents the velocity vector when the UAV passes through the point, *L* is a suitable constant, and for the experiment we take half the distance required for the UAV to gain maximum velocity. Solving Eq. (9) by substituting it into Eqs. (7) and (8) verifies that the trajectory passes through the starting point $C_{1}$ and has velocity $v_{1}$ acceleration $a_{1}$ of zero. Similarly, the target point $C_{n}$ is extended to $c_{n - 1}$, $c_{n}$,$c_{n + 1}$ and $c_{n + 1}$, where $c_{n}$ is the origin $C_{n}$, $c_{n - 1}$ and $c_{n + 1}$ are denoted by Eq. (11).

11 $$\left\{ {\begin{array}{*{20}c} {c_{n - 1} = c_{n} - v_{n} L} \\ {c_{n + 1} = c_{n} + v_{n} L} \\ \end{array} } \right.$$

The solution of the equation also verifies that the trajectory passes through the control point $C_{N}$, the speed of the UAV is $v_{n}$, and the acceleration $a_{n}$ is zero. Therefore as long as we adjust the control points $C_{1}$, $C_{2}$, $C_{3}$, $C_{4}$, …, $C_{N}$, the initialization of the UAV B-spline trajectory can be completed after solving.

In order to quickly initialize the UAV B-spline trajectory, the polynomial fitting method is used for the selection of control points. After obtaining the starting point $C_{1}$ and the ending point $C_{N}$ of the trajectory, the maximum singular value $d_{z}$ between the two points in space is calculated and compared with the set distance $d_{c}$ of the distribution of the control points, to determine whether to add a waypoint. If $d_{z}$ is larger, it is necessary to set waypoints. The expression for the number m of setting waypoints is:

12 $$\left\{ {\begin{array}{*{20}c} {m = [d_{z} /d_{c} ] + 1} & {(d_{z} > d_{c} )} \\ {m = 2} & {d_{z} \le d_{c} } \\ \end{array} } \right.$$

The coordinates of the path point $C_{N}$ are calculated as:

13 $$\left\{ {\begin{array}{*{20}c} {x_{n} = x_{0} (1 - n/m) + \frac{1}{m}x_{N} } \\ {y_{n} = y_{0} (1 - n/m) + \frac{1}{m}y_{N} \,\,\,\,\,\left( {0 < n \le m} \right)} \\ {z_{n} = z_{0} (1 - n/m) + \frac{1}{m}z_{N} } \\ \end{array} } \right.$$

After obtaining each path point it is known that m-1 segments of UAV trajectories are generated, and the polynomial coefficients of each UAV trajectory in the x-axis, y-axis, and z-axis directions, $F_{x} ,F_{y} ,F_{z}$ are solved as shown in Eq. (14).

14 $$\left\{ \begin{gathered} F_{x} = B_{x} /A \hfill \\ F_{y} = B_{y} /A \hfill \\ F_{z} = B_{z} /A \hfill \\ \end{gathered} \right.$$

where, $B_{x}$, $B_{y}$, $B_{z}$ are the parameter vectors in the *x*-axis, *y*-axis, and *z*-axis directions at the beginning and end of each UAV trajectory, and their expressions are:

15 $$\left\{ {\begin{array}{*{20}c} {B_{x} = \left[ {x_{0} ,v_{x0} ,a_{x0} ,x_{n} ,v_{xn} ,a_{xn} } \right]} \\ {B_{y} = \left[ {y_{0} ,v_{y0} ,a_{y0} ,y_{n} ,v_{yn} ,a_{yn} } \right]} \\ {B_{z} = \left[ {z_{0} ,v_{z0} ,a_{z0} ,z_{n} ,v_{zn} ,a_{zn} } \right]} \\ \end{array} } \right.$$

where $x_{0} ,y_{0} ,z_{0} ,v_{x0} ,v_{y0} ,v_{z0} ,a_{x0} ,a_{y0} ,a_{z0}$ are the coordinate, velocity, and acceleration of each segment of the trajectory on the three axes of the starting point. $x_{n} ,y_{n} ,z_{n} ,v_{xn} ,v_{yn} ,v_{zn} ,a_{xn} ,a_{yn} ,a_{zn}$ are the coordinate, velocity, and acceleration of the corresponding segment of the trajectory on the three axes of the ending point. The parameter matrix in Eq. (14) is expressed as:

16 $$A = \left[ {\begin{array}{*{20}c} 0 & 0 & 0 & 0 & 1 & 0 \\ 0 & 0 & 0 & 1 & 0 & 0 \\ 0 & 0 & 2 & 0 & 0 & 0 \\ {t^{5} } & {t^{4} } & {t^{3} } & {t^{2} } & t & 1 \\ {5t^{4} } & {4t^{3} } & {3t^{2} } & {2t} & 1 & 0 \\ {20t^{3} } & {12t^{2} } & {6t} & 2 & 0 & 0 \\ \end{array} } \right]$$

where *t* represents the time required to pass through the corresponding trajectory at the UAV's maximum speed, and the time is set to be twice the time required to fly at the maximum speed, considering that the UAV may not be able to maintain the maximum speed during the start and end segments. $t_{n}$ can be formulated as follows.

17 $$\left\{ \begin{gathered} t_{n} = 2\left( {C_{n + 1} - C_{n} } \right)/v_{\max } \, \left( {n = 0} \right) \hfill \\ t_{n} = \left( {C_{n + 1} - C_{n} } \right)/v_{\max } \, \left( {0 < n < m} \right) \hfill \\ t_{n} = 2\left( {C_{n + 1} - C_{n} } \right)/v_{\max } \, \left( {n = m} \right) \hfill \\ \end{gathered} \right.$$

After obtaining the coordinates of the waypoints and the polynomial coefficients of the m-1 group of coordinates, the polynomial fitting is used to generate the coordinates of the control points, and the solution formula is shown in Eq. (18).

18 $$\left\{ \begin{gathered} x_{n} = \frac{{{\mathbf{K}}_{x} }}{{\mathbf{C}}} \hfill \\ y_{n} = \frac{{{\mathbf{K}}_{y} }}{{\mathbf{C}}} \hfill \\ z_{n} = \frac{{{\mathbf{K}}_{z} }}{{\mathbf{C}}} \hfill \\ \end{gathered} \right.$$

where ${\mathbf{C}}$ is the coefficient matrix associated with the number of waypoints and ${\mathbf{K}}_{x}$, ${\mathbf{K}}_{y}$, ${\mathbf{K}}_{z}$ are the augmentation vector of the coefficient vector $F_{x} ,F_{y} ,F_{z}$ of the polynomial. After obtaining the control points according to Eq. (3), the path initialization is completed.

The trajectory initialization process has only the starting point and the endpoint of the trajectory, and to facilitate the trajectory solving, the initialization process of the trajectory is calculated using the parameter *S*, but the trajectory optimization process is more convenient to use the time parameter t to solve the trajectory. The conversion formula for the parameters *S* and t of the UAV trajectory is:

19 $$P\left( s \right) = \left[ \begin{gathered} x(s) \hfill \\ y(s) \hfill \\ z(s) \hfill \\ \end{gathered} \right] = P\left( t \right) = \left[ \begin{gathered} x(t) \hfill \\ y(t) \hfill \\ z(t) \hfill \\ \end{gathered} \right]$$

20 $$\frac{dP\left( t \right)}{{dt}} = \frac{\partial P\left( s \right)}{{\partial s}}\overline{s} \Rightarrow \left[ \begin{gathered} v_{x} (t) \hfill \\ v_{y} (t) \hfill \\ v_{z} (t) \hfill \\ \end{gathered} \right] = \left[ \begin{gathered} v_{x} (s) \hfill \\ v_{y} (s) \hfill \\ v_{z} (s) \hfill \\ \end{gathered} \right]\overline{s}(t)$$

## Optimization of trajectory

### Clamped B-splines

According to de Boor-Cor's recursive formula (21), it can be shown that each higher-order B-spline is a convex linear combination of two lower first-order B-splines. Each segment of the B-spline curve must have enough basis functions to match the control points if the interval is legal. Therefore, in the proposed algorithm, *n* + 4 node vectors $\left( {u_{0} , \cdots ,u_{n + 3} } \right)$ are required if there are n control points. Four nodes $\left( {u_{i} ,u_{i + 1} ,u_{i + 2} ,u_{i + 3} } \right)$ are needed if one wants to determine a 4th-order B-spline $P_{i}$. Neighboring B-splines have 2 control points that are the same to ensure that the splicing between every two segments is good.

21 $$\left\{ \begin{gathered} B_{i,1} \left( u \right) = \left\{ \begin{gathered} 1 \, u_{i} < u_{i + 1} \hfill \\ 0{\text{ otherwise }} \hfill \\ \end{gathered} \right. \hfill \\ B_{i,k} = \frac{{u - u_{i} }}{{u_{i + k - 1} - u_{i} }}B_{i,k - 1} \left( u \right) + \frac{{u_{i + k} - u}}{{u_{i + k} - u_{i + 1} }}B_{i + 1,k - 1} \left( u \right) \hfill \\ \end{gathered} \right.$$

**Figure 1:**
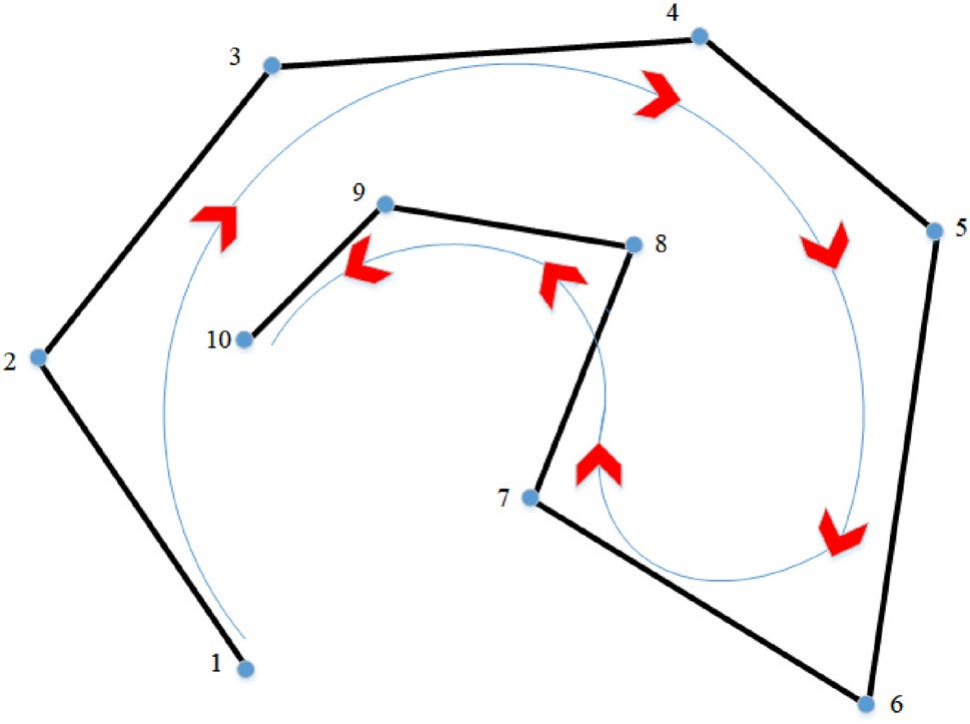
Structure of clamped B-spline.

The UAV trajectory initialization process, by assuming the increase of control points to ensure that the UAV passes through the trajectory start point, the solution passes through is the beginning and end of the two points of the velocity of $v_{1}$ and $v_{n}$, respectively. However, the speed of the UAV in the beginning two points can only be zero. Therefore, in the trajectory solution process outlined in this paper, the first four and the last four node vectors remain consistent. This B-spline is called clamped B-spline and its structure is shown in Fig. 1.

The graph features 10 control points and 14 nodes with node vectors [0, 0, 0, 0, 0, 0.125, 0.25, 0.375, 0.5, 0.625, 0.75, 0.875, 1, 1, 1, 1]^T^. Figure 1 perfectly illustrates the two main properties of the clamped B-spline. The curve passes through the head and tail control points and has a strong convex hull^25,26^. Strong convex hull property means that if the node vectors $u$ are located in $\left[ {u_{i} ,u_{i + 1} } \right]$, then the curve $P(u)$ is located within the convex hull defined by control points $C_{i} ,\,C_{i - 1} ,\,C_{i - 2} ,\,C_{i - 3}$ $\left( {i \ge 3} \right)$, which indirectly verifies that a control point only affects local trajectories. The B-spline function is localized, and this property can solve the problem of local changes in the trajectory affecting the overall trajectory planning when the UAV encounters an obstacle. The collision part of the trajectory is changed by adjusting the relevant control points, while the convex hull of the B-spline ensures that the trajectory meets the restricted range.

### Obstacle avoidance optimization

**Figure 2:**
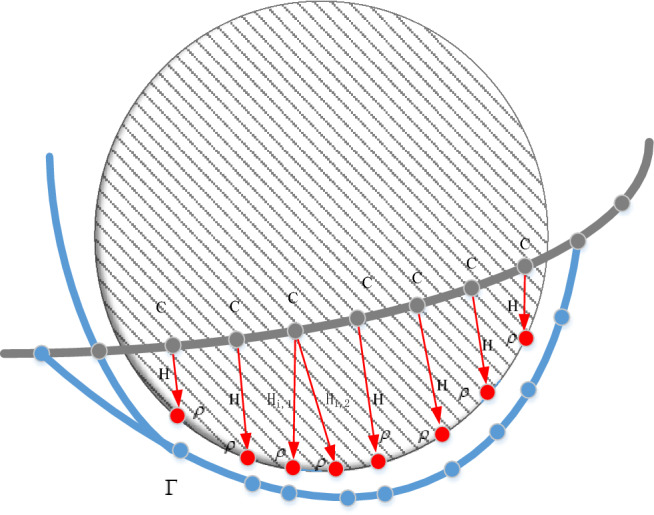
Trajectory obstacle avoidance control point variation diagram.

When a trajectory collides with an obstacle, the control point of the collision is selected, and the corresponding point of the control point is generated on the surface of the obstacle. Each control point $C_{i}$ may have more than one corresponding point $\rho_{i,j}$ on the obstacle surface, but each corresponding point $\rho_{i,j}$ belongs to only one control point. According to the corresponding repulsive direction vector $H_{i,j}$ generated by the positions and $\rho_{i,j}$, the relationship between the three is shown in Fig. 2. *i* coincide with the index of the original control point, and j is the index of the corresponding point and the opposite direction vector. The distance between the obstacle surface point and the original control point is calculated as shown in Eq. (22).

22 $$D_{i,j} = \left( {C_{i} - \rho_{{_{i,j} }} } \right) \cdot H_{i,j}$$

Since the trajectory is continuously collision detected and optimized, to avoid the resulting duplicates of $H_{i,j}$ and $\rho_{i,j}$, we consider obstacles encountered by the control points $C_{i}$ as newly discovered obstacles only by restricting $D_{i,j}$ > 0. This restriction helps us to find obstacles that affect the new trajectory, reducing the amount of computation that can be done to update the new trajectory faster.

Environment as an important reference factor in UAV obstacle avoidance algorithms, a large amount of environmental data must be stored as a prerequisite for obstacle avoidance, and at the same time, appropriate trajectory algorithms are established to avoid obstacles. This means that there is a large amount of data to be computationally processed, which is undoubtedly an insurmountable problem for the arithmetic power of micro UAVs. The article algorithm only needs to store the environmental data in the narrow space near the B-spline trajectory, while the repulsive force algorithm is simple and efficient, which greatly reduces the amount of computation^27^. Moreover, because it does not require a lot of environmental information, it avoids the problem of UAVs falling into local minima and does not require collision-free trajectories to be generated in advance.

**Figure 3:**
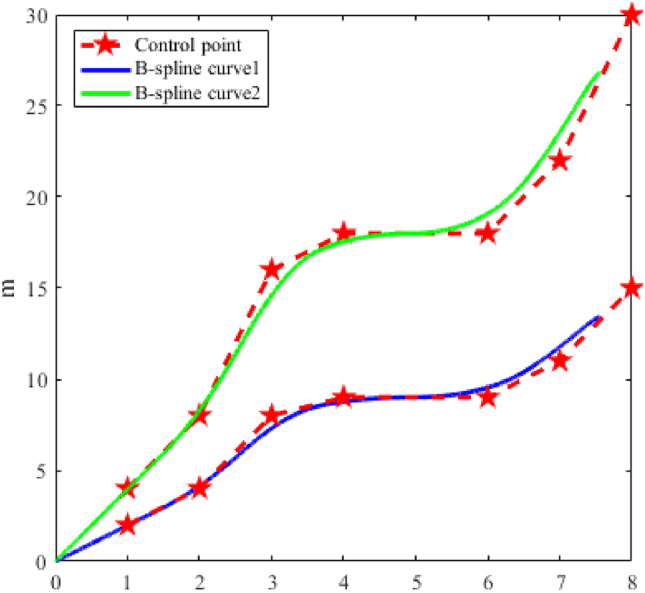
Change of B-spline curve by control points selection.

Since the trajectory is generated based on the B-spline curve, it is affected by the selection of basis function and control point, the B-spline curve structure is shown in Fig. 3. The distance between control points has a great influence on trajectory generation. If the distance between the trajectory control points is too large, the trajectory deformation caused by each iteration of the trajectory will be large, and unnecessary UAV performance waste will occur when facing small obstacles. If the distance between control points is too small, more iterative calculations are needed and even failed to planning when facing large obstacles.

### Gradient-based trajectory optimization

According to Eq. (12) it can be seen that in the B-spline parameterization process, we use a uniform B-spline so that the time interval $\Delta t$ in the control points is the same and very small, so the UAV velocity $v_{i}$, acceleration $a_{i}$, and jerk $j_{i}$ between the two control points can be expressed as:

23 $$\left\{ \begin{gathered} v_{i} { = }\frac{{C_{i + 1} - C_{i} }}{\Delta t} \hfill \\ a_{i} = \frac{{v_{i + 1} - v_{i} }}{\Delta t} \hfill \\ j_{i} = \frac{{a_{i + 1} - a_{i} }}{\Delta t} \hfill \\ \end{gathered} \right.$$

The trajectory planning task for UAVs is carried out in a differentially flat space with set control points $C_{i}$, $C_{i} \in R^{3}$, so the trajectory optimization process transforms into:

24 $$J = \lambda_{s} J_{s} + \lambda_{c} J_{c} + \lambda_{f} J_{f}$$

where $J_{s}$ is the smoothness penalty, $J_{c}$ represents the collision penalty, $J_{f}$ represents the feasibility penalty, $\lambda_{s}$, $\lambda_{c}$ and $\lambda_{f}$ are the weights of the three penalties.

Smoothness penalty: the smoothness penalty is formulated as the curvature of the trajectory, the smoothness of the trajectory is judged by the geometric information of the human–computer trajectory, if the higher curvature of the trajectory represents more difficulty for the UAV to track the trajectory. Therefore, the functional expression of the smoothing penalty is:

25 $$J_{s} = \sum\limits_{i = 0}^{n} {\sqrt {{{\left| {a_{i} } \right|^{2} \left| {v_{i} } \right|^{2} - (v_{i} \cdot a_{i} )^{2} } \mathord{\left/ {\vphantom {{\left| {a_{i} } \right|^{2} \left| {v_{i} } \right|^{2} - (v_{i} \cdot a_{i} )^{2} } {\left| {v_{i} } \right|}}} \right. \kern-0pt} {\left| {v_{i} } \right|}}^{3} } }$$

Minimizing each control point acceleration $a_{i}$ and jerk $j_{i}$, makes the whole trajectory smooth.

Collision penalties: the purpose of establishing collision penalties is to keep the UAV away from obstacles and keep the two at a safe distance $s_{f}$. A segmentation function is established to determine the rate of change of the collision penalty based on the size of $D_{i,j}$. Therefore, the expression of the collision penalty function for the corresponding point $\rho_{i,j}$ of each control point is:

26 $$\begin{gathered} j_{c} (i,j) = \left\{ \begin{gathered} 0 \, \left( {w_{i,j} \le {0}} \right) \, \hfill \\ w_{i,j}^{3} \, \left( {0 < w_{i,j} \le s_{f} } \right) \hfill \\ 3s_{f} w_{i,j}^{2} - 3s_{f}^{2} c_{i,j} + 3s_{f}^{3} \, \left( {w_{i,j} > s_{f} } \right) \hfill \\ \end{gathered} \right. \hfill \\ w_{i,j} = s_{f} - D_{i,j} \hfill \\ \end{gathered}$$

where $j_{c} (i,j)$ is the cost of generating the repulsive direction vector $H_{i,j}$ at the corresponding point $\rho_{i,j}$ on the control point $C_{i}$. The cost of each control point is calculated independently, and when a control point generates multiple repulsive direction vectors, it represents an increase in the cost of obstacle avoidance. Thus, the collision penalty incurred by a control point is calculated as:

27 $$J_{c} \left( i \right){ = }\sum\limits_{j = 1}^{e} {j_{c} (i,j)}$$

where $e$ is the number of control points generating repulsive direction vectors ${\mathbf{H}}$. The cost of a UAV trajectory collision is the accumulation of the collision penalty $J_{c} \left( i \right)$ for all control points. Therefore, the total cost of the collision penalty is:

28 $$J_{c} \left( i \right){ = }\sum\limits_{j = 1}^{e} {j_{c} (i,j)}$$

To obtain the total cost of collision $J_{c}$, we obtain the gradient by deriving it with the expression:

29 $$\frac{{\partial J_{c} }}{{\partial C_{i} }} = \sum\limits_{i = 1}^{n} {\sum\limits_{j = 1}^{e} {H_{i,j} } \left\{ \begin{gathered} 0 \, \left( {w_{i,j} \le {0}} \right) \hfill \\ - 3w_{i,j}^{2} \, \left( {0 < w_{i,j} \le s_{f} } \right) \hfill \\ - 6s_{f} w_{i,j} + 3s_{f}^{2} \, \left( {w_{i,j} > s_{f} } \right) \hfill \\ \end{gathered} \right.}$$

Feasibility penalty: we need to constrain the UAV trajectory in *x*, *y*, and *z* directions to ensure that the speed, acceleration, and jerk of the UAV in each dimension satisfy the UAV performance constraints. Due to the convex envelopment of the B-spline, we can restrict the UAV trajectory by restricting the derivatives of the control points.

30 $$J_{f} = \sum\limits_{i = 1}^{n} {w_{v} F(v_{i} ) + \sum\limits_{i = 1}^{n - 1} {w_{a} F(a_{i} ) + \sum\limits_{i = 1}^{n - 2} {w_{j} F(j_{i} } } } )$$

where $w_{v}$, $w_{a}$ and $w_{j}$ are the weights of the feasibility penalty in terms of velocity, acceleration and jerk. $F$ is a quadratically continuous differentiable function of the higher-order derivatives of the control points.

31 $$F(c) = \sum\limits_{r = x,y,z} {f(c_{r} )}$$

32 $$f(c_{r} ) = \left\{ \begin{gathered} a_{1} c_{r}^{2} + b_{1} c_{r} + c_{1} \, (c_{r} \le - c_{j} ) \hfill \\ ( - \lambda c_{m} - c_{r} )^{3} \, ( - c_{j} < c_{r} < - \lambda c_{m} ) \hfill \\ 0 \, ( - \lambda c_{m} \le c_{r} \le \lambda c_{m} ) \hfill \\ (c_{r} - \lambda c_{m} )^{3} \, (\lambda c_{m} < c_{r} \le c_{j} ) \hfill \\ a_{2} c_{r}^{2} + b_{2} c_{r} + c_{2} \, ( \, c_{r} \ge c_{j} ) \, \hfill \\ \end{gathered} \right.$$

where $c_{r} \in C \in \left\{ {v_{i} ,a_{i} ,j_{i} } \right\}$, $a_{1}$, $b_{1}$, $c_{1} \,$, $a_{2}$, $b_{2}$ and $c_{2}$ are able to satisfy the second-order continuity of the function $F$. $c_{m}$ is the limit of the derivatives, $c_{j}$ is the node of the second and third intervals. $\lambda$ is the elasticity coefficient and $0 < \lambda < 1$ to make the final result satisfy the constraints.

### Time adjustment

The time $T_{i}$ of an individual trajectory segment $P_{i}$ is mainly determined by the number and position of control points. In the initialization phase of the trajectory, we assign times to individual segments. When the UAV changes its path due to an obstacle, the speed $V_{i}$, acceleration $A_{i}$ and jerk $J_{i}$ of the UAV exceed the UAV's own limits, needs to adjust the time, the expression for the time conversion ratio is:

33 $$u = \max \left\{ {\left| {v_{m} {/}V_{i} } \right|,\sqrt {\left| {a_{m} {/}A_{i} } \right|} ,\sqrt[3]{{\left| {j_{m} {/}J_{i} } \right|}}} \right\}$$

where $v_{m}$, $a_{m}$ and $j_{m}$ respectively represent maximum speed, acceleration, and jerk on the *x*, *y*, and *z-*axis. The time for the UAV to complete the reassignment of the path fragments is as follows:

34 $$T_{i}{\prime} = uT_{i}$$

### Numerical solution

This paper proposes the use of gradient for trajectory optimization, which can be seen as solving extreme value problems of multivariate objective functions^28^. The optimal approach for solving such unconstrained optimization problems is to use the Newton method. By utilizing the solved data during the objective function generation process, the requirement for the fast restart of trajectory computation can be met, and repeated computation can be avoided, thereby improving the solving speed. However, solving the UAV trajectory requires extremely high real-time performance. In the solving process of Newton's method, the inverse of the Hessian matrix needs to be computed, which exceeds the computational power of the UAV. Therefore, the proposed quasi-Newton algorithm is used for solving^29–31^.

There are various quasi-Newton algorithms, and in this paper, the L-BFGS algorithm is utilized^32–34^. This algorithm ensures a high success rate in obstacle avoidance and also exhibits good performance in terms of solving accuracy and restart loss. The solution process is as follows, $x \in R^{3}$, $f(x)$ and the update formula for *x* is:

35 $$x_{k + 1} = x_{k} - \lambda H_{k}^{ - 1} \nabla f_{k}$$

where *λ* is the learning rate and the iterative formula for $H_{K}$ is:

36 $$H_{k + 1} = V_{k}^{T} H_{k} V_{K} + \rho_{k} s_{k} s_{k}^{T}$$

where $\rho_{k} {\text{ = (y}}_{k}^{T} s_{k} )^{ - 1}$, $v_{k} = I - \rho_{k} {\text{y}}_{k} s_{k}^{T}$, $s_{k} = x_{k + 1} - x_{k}$, $y_{k} = \nabla f_{k + 1} - \nabla f_{k}$. $H_{K}$ also does not need to be solved explicitly, and $H_{K}$ is approximated by $B_{k}$. Thus, the proposed Newtonian condition is:

37 $$B_{k + 1} s_{k} = y_{k}$$

38 $$B_{k + 1} = B_{k} + P_{k} + Q_{k}$$

Make $P_{k}$ and $Q_{k}$ satisfy:

39 $$\left\{ \begin{gathered} P_{k} s_{k} { = }y_{k} \hfill \\ Q_{k} s_{k} = - B_{k} s_{k} \hfill \\ \end{gathered} \right.$$

The iterative formula for the algorithmic matrix $B_{k + 1}$ can be obtained after obtaining the appropriate $P_{k}$ and $Q_{k}$:

40 $$B_{k + 1} = B_{k} + \frac{{y_{k} y_{k}^{T} }}{{y_{k}^{T} s_{k} }} - \frac{{B_{k} s_{k} s_{k}^{T} B_{k} }}{{s_{k}^{T} B_{k} s_{k} }}$$

The L-BFGS algorithm requires $H_{k}^{0}$ to be a positive definite matrix in order to ensure gradient descent. To achieve convergence, a monotone line search is performed under strong Wolfe conditions, with the Hessian matrix $H_{k}^{0}$ being used.

41 $$H_{k}^{0} = \frac{{s_{k - 1}^{T} y_{k - 1} }}{{y_{k - 1}^{T} y_{k - 1} }}I$$

## Experiment results and analysis

Utilizing the ROS platform^35^, we conducted UAV autonomous flight experiments across various simulated environments and compared them with other algorithms to assess the proposed algorithm's effectiveness. During the experiment, the UAV faced limitations in acquiring global obstacle information. When approaching an obstacle, the UAV was able to gather local obstacle information through a simulated camera. This information may not even be all the information about an obstacle.

**Figure 4:**
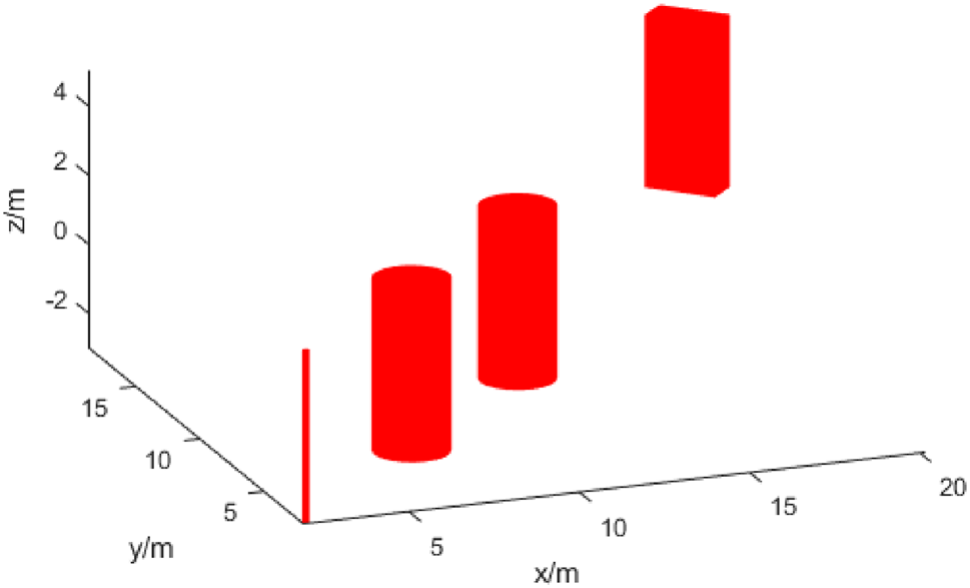
Simple experimental environment.

Initially, the simulation experiment was conducted in a setting with simple obstacles. In experiments, the UAV flew from an initial point to a target point at a constant speed and stable attitude, while traversing an area with four obstacles. Four obstacles were distributed within the simulation map of [25.0 m, 25.0 m, 5.0 m], consisting of three cylinders with varying thicknesses and a wall. Experiments can intuitively reflect the trajectory generation process of UAVs. When the trajectory generated in the previous iteration fails to avoid obstacles, the proposed algorithm can quickly generate a new path. The experimental environment is depicted in Fig. 4.

**Figure 5:**
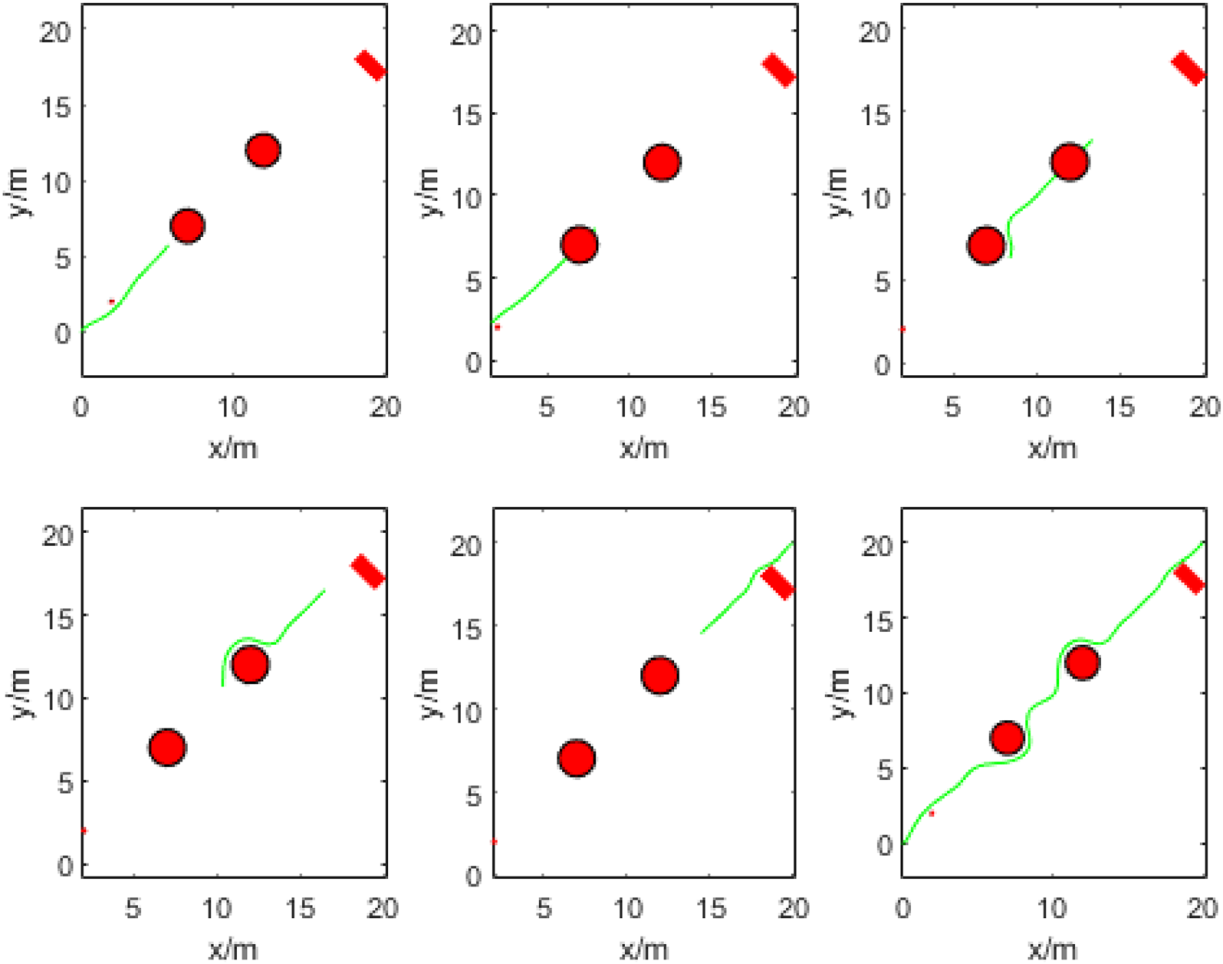
Path generation process in a simple obstacle environment.

**Figure 6:**
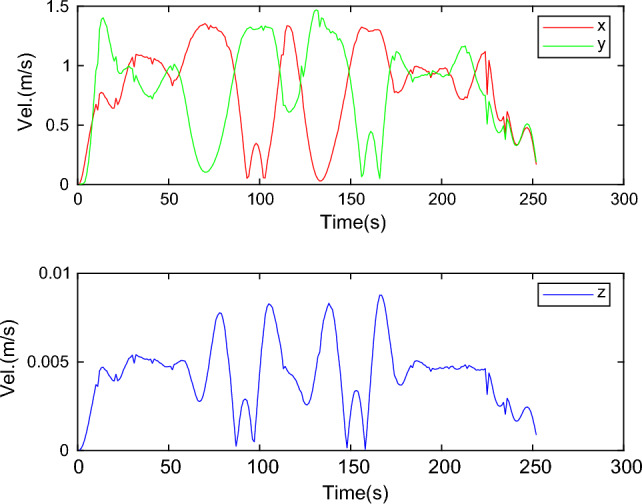
UAV three-axis speed in simple obstacle environment.

The UAV trajectory is generated in segments, without considering collision during the initialization process. The B-spline order is 3, resulting in approximately 25 control points per segment of the trajectory. Each segment has a horizontal length of around 7 m, with an initial distance interval of about 0.3 m between neighboring points. The safety distance between the UAV and obstacles is set at 0.8 m. These parameters are considered optimal as they effectively balance the degrees of freedom and complexity of the UAV trajectory. Experiment 1 involves generating the UAV running trajectory using the B-spline algorithm in a simple obstacle environment. The total length of the trajectory is 30.956 m. The process of UAV running obstacle avoidance is illustrated in Fig. 5, and the UAV’s three-axis speed is shown in Fig. 6.

**Figure 7:**
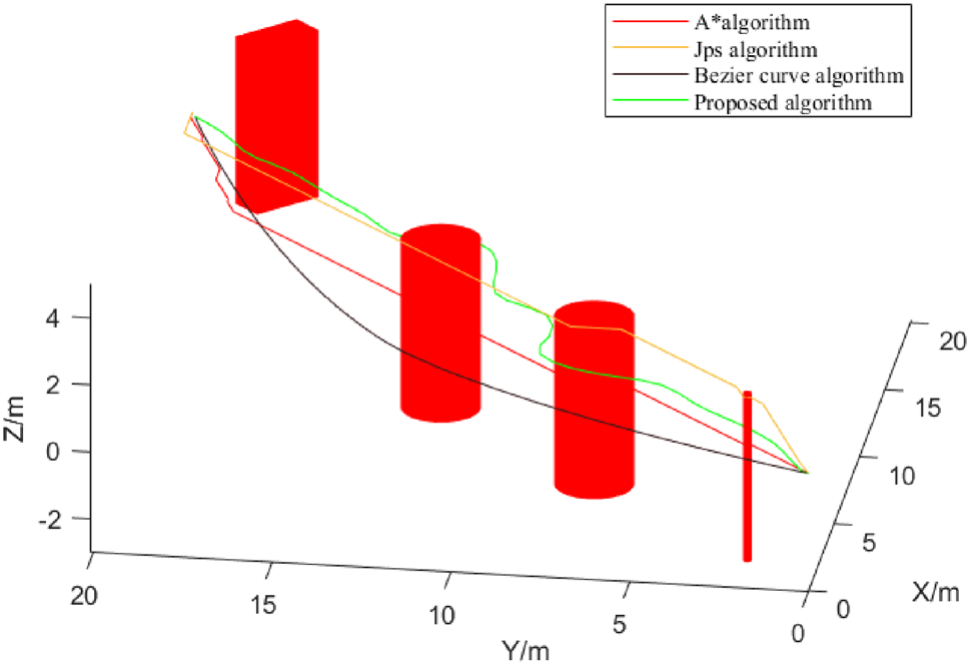
Comparison of trajectories in Experiment 1.

In this paper, the proposed path planning algorithm is compared with two traditional graph search algorithms, A* and Jump Point Search (JPS), as well as the Bezier curve fitting algorithm. Three algorithms require sensing most of the obstacles in the environment in order to discover the optimized path. The article algorithm focuses more on path optimization compared to the other three algorithms, particularly in a simple environment, resulting in smaller data processing. The paths generated by the three algorithms in the identical experimental environment are compared, and the results are shown in Fig. 7.

Based on the findings presented in Fig. 7 and Table 1, it can be observed that the A* and JPS algorithms demonstrate better performance in terms of time efficiency in simple environments. This is mainly because the obstacle environment is relatively simple, and the data processing amount is too small. When faced with more complex environments, the advantages of algorithms will be shown. Meanwhile, these algorithms are not as effective as the algorithm proposed in the paper in terms of the trajectory length and the number of nodes used. On the other hand, the B-spline trajectory planning method algorithm and Bezier curve algorithm generate smoother trajectories that are well-suited for UAV dynamics and can be easily implemented in real-life scenarios.

**Table 1:** Path comparison of the three algorithms.

Algorithms	Lengths (m)	Time (s)	Nodes used
A*	34.705	20.15	1540
JPS	33.529	19.29	3126
Bezier curve	31.572	20.52	1728
Proposed	30.956	20.32	236

**Figure 8:**
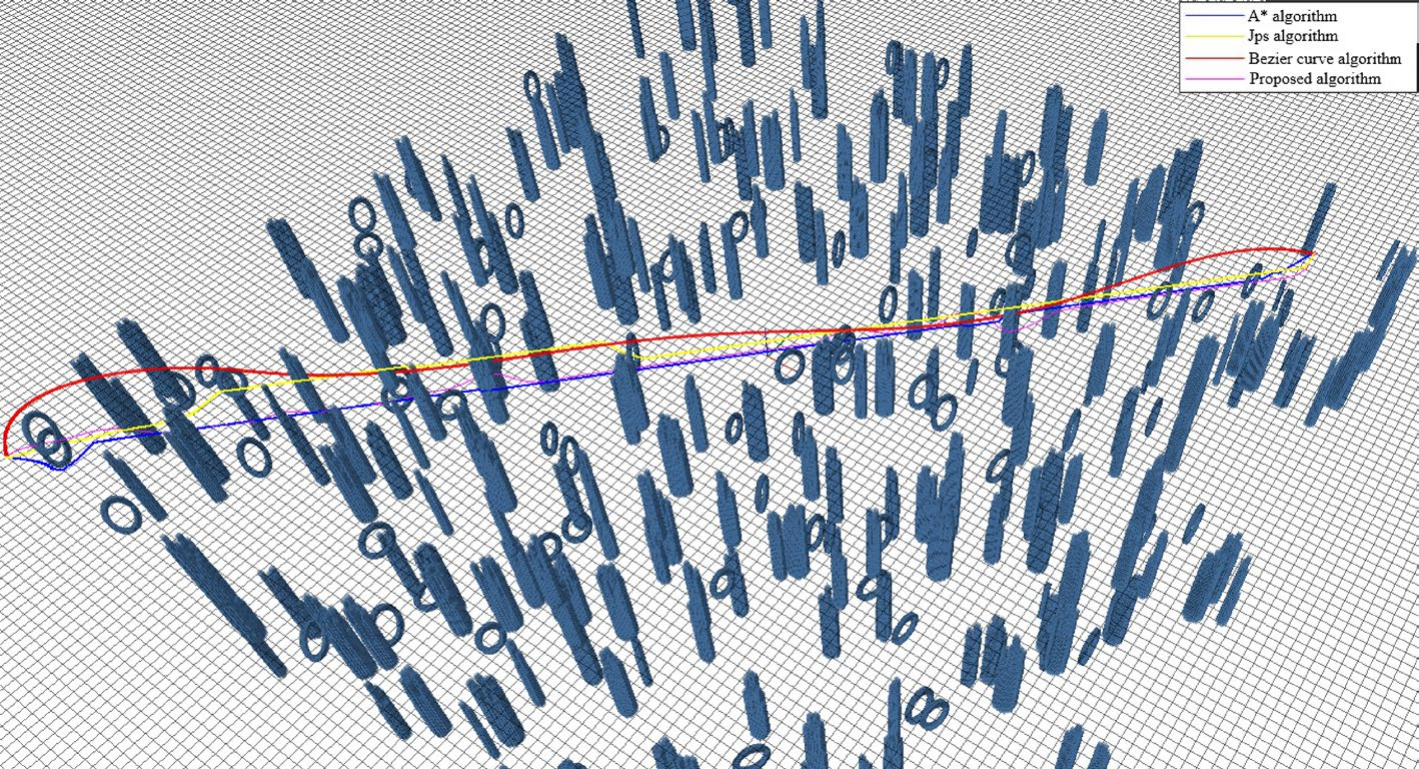
Comparison of trajectories in Experiment 2.

To further validate the effectiveness of the algorithm proposed in this paper, Experiment 2 randomly generated 300 cylindrical obstacles and Circular obstacles within a square range of 50 m on each side. The position, radius, and height of each cylindrical obstacle were randomly generated, and there was no overlap between the obstacles. Three path planning methods operate independently in the same environment mentioned above. The UAV initiates its flight from the coordinates [− 25, − 25, 5] and navigates towards the final destination point^5, 25^. The resulting trajectories generated by the UAV using the three trajectory calculation methods are illustrated in Fig. 8.

The results presented in Fig. 8 and Table 2 indicate that the proposed algorithm effectively tackles real-time obstacle avoidance in randomized and complex environments. While the A*, JPS, and Bezier curve algorithms also demonstrate success in solving the obstacle avoidance problem during the experiment, they struggle to handle the significant amount of data generated by more complex environments, leading to suboptimal solutions. This highlights the superior stability of the algorithms proposed in this paper. The trajectory shape in Fig. 8 demonstrates that the proposed algorithm in the paper primarily achieves obstacle avoidance through in-plane roundabouts. This approach ensures the stability of the UAV while increasing the solving speed and generating a smoother UAV trajectory.

**Table 2:** Comparison of paths in complex environments.

Algorithms	Lengths (m)	Time (s)	Nodes used
A*	73.24	42.32	3751
JPS	72.85	41.25	23,466
Bezier curve	73.59	42.68	3962
Proposed	72.30	40.11	952

**Figure 9:**
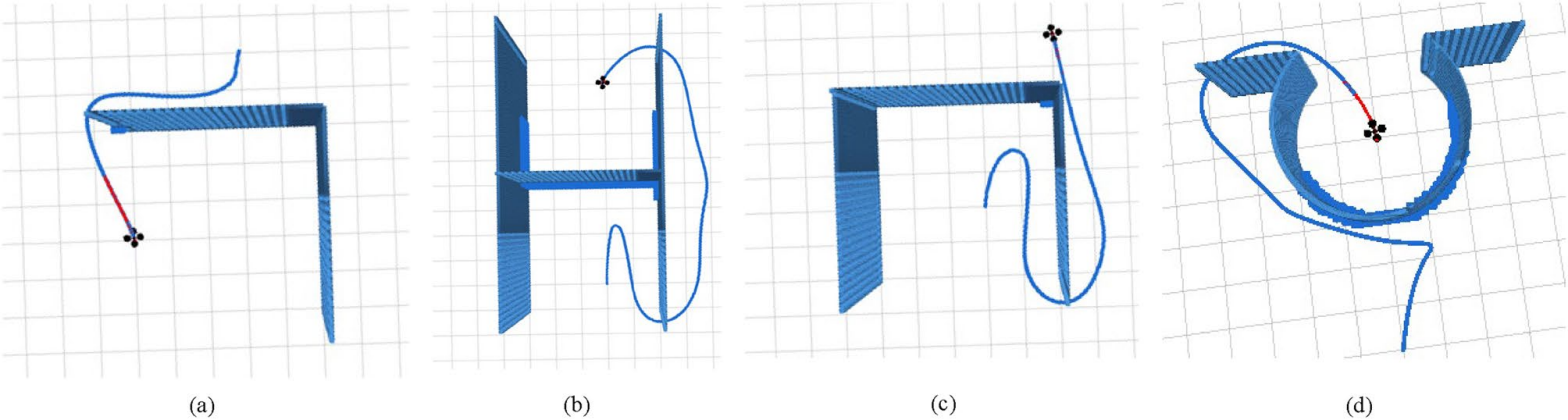
Obstacle avoidance trajectory of UAV in special obstacle environments: (**a**) L-shape obstacle; (**b**) H-shape obstacle; (**c**) U-shape obstacle; (**d**) Omega shape obstacle.

In addition, we conducted the autonomous flight obstacle avoidance simulation experiment of UAVs in L, H, U, and omega under four special obstacle environments, and the generated UAV trajectories are shown in Fig. 9. In these cases, the algorithm still provides a safe and smooth UAV trajectory that allows the UAV to traverse these obstacles.

When the UAV passes L-shaped obstacles, a smooth trajectory is generated, as shown in Fig. 9a. The UAV initially fits the control points by polynomial and connects the control points to generate the trajectory. When no obstacle is found, the trajectory flies along the line between the starting point and the destination place, as shown in Fig. 9b,c, which may cause the UAV to waste kinetic energy. Of course, this is limited by the UAV's ability to sense obstacles, and similar situations can be avoided by carrying better sensors. Secondly, when the algorithm pursued smoothness, the UAV circled a part of the distance, as shown in Fig. 9c,d. However, the UAV is easier to complete the smoother trajectories than the sharp ones without requiring global obstacle information, and it has higher adaptability to the unknown environment.

**Figure 10:**
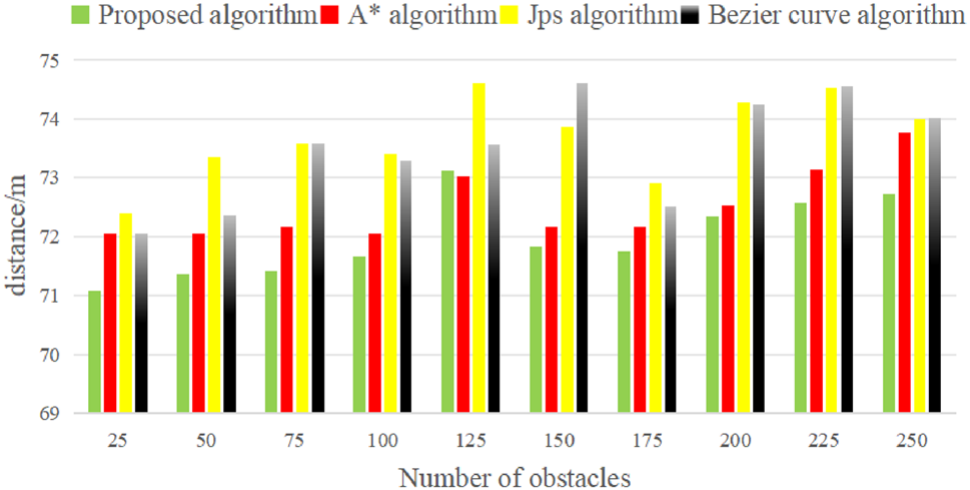
Comparison of UAV trajectory lengths.

Under the same conditions of obstacle generation methods, boundary constraints, and route constraints, the four algorithms operate independently in environments with ten different numbers of obstacles. The comparison of trajectory lengths generated by the four algorithms is presented in Fig. 10. In 10 obstacle density environments, the average trajectory lengths for the four algorithms are 71.98 m, 72.50 m, 73.69 m, and 73.47 m respectively. The comparison in Fig. 10 reveals that the proposed algorithm exhibits a shorter trajectory and runtime.

## Conclusion

This paper focuses on developing a B-spline curve-based UAV path planner to achieve a smoother and more suitable flight path. The proposed algorithm demonstrates comparable performance in planning distance and time to other advanced path planning algorithms while producing a trajectory that is better suited for UAV flight. In addition, the algorithm only requires obstacle information in the narrow space around the trajectory, leading to reduced data processing and enabling the UAV to navigate through unknown obstacle environments swiftly and safely. Simulation experiments confirmed the effectiveness and reliability of the algorithm.

## Data Availability

The data that support the findings of this study are available on request from the corresponding author, Pengxiang Sun, upon reasonable request.
